# Effect of bladder volume on dose of exposure to dangerous organs and incidence of cystitis and enteritis in patients with cervical cancer after external radiotherapy

**DOI:** 10.3389/fonc.2026.1760076

**Published:** 2026-03-20

**Authors:** Yanjiao Wu, Xiaoying Xue, Linlin Su, Yibin Liu

**Affiliations:** 1 Department of Radiotherapy, The Second Hospital of Hebei Medical University, Shijiazhuang, Hebei, China; 2 Department of Gynecology, The Second Hospital of Hebei Medical University, Shijiazhuang, Hebei, China

**Keywords:** bladder volume, cervical cancer, external pelvic irradiation, organ at risk, real-world data

## Abstract

**Background and objective:**

This study looked into how bladder volume affected clinical outcomes in individuals with cervical cancer after external radiation therapy as well as the radiation dosage received by organs at risk.

**Methods:**

A retrospective cohort analysis was carried out on 142 patients with cervical cancer who underwent postoperative radiotherapy between January 2017 and September 2020. Based on differences in bladder capacities, these patients were divided into three groups: Group A (V ≤ 300 ml), Group B (300<V ≤ 500 ml), and Group C (V>500 ml). The purpose of the study was to determine how different bladder volumes affected the radiation doses that the rectum (V30, V40, V50, D2cm^3^), small intestine (V30, V40, V50, D2cm^3^), and bladder (V30, V40, V50, D2cm^3^) got.

**Key findings and limitations:**

The median age was 53 years, and the usual follow-up period was 48 months. Group A had considerably higher values for bladder V30 and V40 (73.83 ± 11.56% and 47.21 ± 6.953%, respectively; *p* = 0.0130 and *p* = 0.0033). The results of the V40 measurement in the small intestine were also highest in group A (37.79 ± 10.66%; *p* = 0.0151), while in the bladder and rectum (57.85 ± 4.042 Gy, 51.35 ± 3.627 Gy; *p* = 0.0006 and *p* = 0.0211, respectively), group C had the highest values. Among the groups, group B exhibited a notably decreased incidence of radiation cystitis (*p* = 0.0396).

**Conclusions and clinical implications:**

For patients with cervical cancer undergoing external irradiation, a bladder volume of 300ml to 500ml is ideal.

**Advancing practice: what does the study add?:**

This study identifies an optimal bladder volume range of 300–500 mL during external beam radiotherapy for cervical cancer. Compared to smaller or larger volumes, this range reduces radiation exposure to critical organs, leading to fewer side effects. These findings provide practical, evidence-based guidance for bladder filling protocols to improve patient outcomes.

**Patient summary:**

For patients receiving radiation therapy for cervical cancer, keeping the bladder moderately full (about 300–500 mL) during treatment may help protect the bladder and bowel from radiation damage. This simple measure was linked to fewer mild side effects, such as bladder irritation or diarrhea. Patients can discuss with their doctors how to maintain a comfortable bladder volume to potentially improve their quality of life during and after treatment.

## 1 Introduction

Women’s health is seriously threatened by cervical cancer, a common clinical gynecological cancer that primarily affects developing nations and is trending toward younger age groups (1, 2). The main treatment option for cervical cancer, as per the guidelines of the National Comprehensive Cancer Network (NCCN), is radiotherapy, which can be administered to patients at any stage of the disease (3).

The incidence of radiation-induced proctitis and cystitis depends on the dose given as well as the physical state of the patient (4). Treatment options for cervical cancer include intensity-modulated radiation therapy (IMRT) and spiral tomography (TOMO), which reduce radiation exposure to adjacent organs while providing sufficient coverage of the target area to meet therapeutic needs (5–9). Changing the volume of the bladder is a relatively controllable element that influences the dosage of radiation therapy and offers a new way to avoid radiation-related problems.

In addition to being correlated with patients undergoing IMRT and TOMO treatments, the degree of bladder filling can also have a substantial impact on the development of radiation-induced cystitis and proctitis. These characteristics can vary greatly across individuals. While many comparable reports from both domestic and international sources (10, 11) have examined the effects of varying bladder filling volumes on patients’ organs at risk, no study has explicitly examined the ideal bladder filling volume or established pertinent guidelines for clinical practice. The best bladder filling volume must still be determined, but studies have shown that it is possible to assess bladder volume using B-ultrasonography and other tools prior to positioning and treatment for patients with cervical cancer, thanks to technological advancements in the field (12). However, it’s crucial to ascertain the proper bladder filling volume initially. Thus, the purpose of this study is to determine the proper bladder filling volume for cervical cancer radiation therapy.

## 2 Patients and methods

The current clinical investigation was approved by the Ethics Committee of Hebei Medical University’s Second Hospital (2021-P041). The trial was carried out in compliance with the guidelines provided in the Declaration of Helsinki and established protocols for human clinical studies. The requirement for informed consent was waived because only de-identified data were analyzed and no direct patient contact occurred.

### 2.1 Patients

Inclusion criteria: Inclusion criteria: 1. Confirmed diagnosis of cervical squamous cell carcinoma; 2. Initial pelvic radiotherapy treatment; 3. Completion of full course of radiotherapy; 4. Karnofsky Performance Status (KPS) score ≥80 points; 5. Absence of prior malignant tumors; 6. No contraindications or allergies to CT/MRI scans and corresponding contrast agents.

Exclusion criteria: 1. Pathologically classified non-squamous cell carcinoma of the cervical region; 2. Previous pelvic radiation therapy; 3. Withdrawal from treatment; 4. Karnofsky Performance Status (KPS) score <80 points; 5. Presence of another tumor history; 6. History of contraindications to CT/MRI scanning or allergy to corresponding contrast media, without prior intensive scanning.

### 2.2 Treatment procedures and research steps

1) An hour before the CT scan, the bladder and rectum were evacuated, and 500 milliliters of filtered water were then given over the course of 20 minutes. The patient was then placed in a supine position and fastened with a thermoplastic body membrane.

2) Make the most of the CT simulator to streamline the scanning procedure, accurately define the target area by combining CT and MR pictures. The clinical target volumes were contoured by integrating relevant Radiation Therapy Oncology Group (RTOG) consensus guidelines with established institutional gynecological radiotherapy protocols (13). Subsequently, a 7-mm isotropic margin was applied to generate the planning target volume, compensating for setup uncertainties and organ motion. Based on this, a detailed radiation plan was then created.

1) Extracorporeal pelvic irradiation was administered to all patients along with concomitant platinum-based chemotherapy and intracavitary brachytherapy. Using the Swedish Medical Company’s Synergy electron linear accelerator, extracorporeal pelvic irradiation was administered using either the TomoTherapy treatment planning system for spiral tomography with a 6MVX photon line comprising nine fields or Simultaneous Integrated Boost Intensive-Modulated Radiation Therapy (SIB-IMRT). All patients received pelvic external radiotherapy to a dose of 50.4 Gy in 28 fractions. The total treatment regimen was then tailored according to treatment intent: patients in the radical radiation group received a high-dose-rate brachytherapy (HDR-BT) boost of 30 Gy in 5 fractions to point A, while patients in the postoperative radiation group received a HDR-BT boost of 12 Gy in 2 fractions prescribed to 0.5 cm below the vaginal mucosa. Residual or high-risk pelvic lymph node metastases (PGTVnd) in both groups received a simultaneous integrated boost of 59.92 Gy in 28 fractions during EBRT. In accordance with RTOG guidelines, areas and dose restrictions pertaining to threatened organs were established. The bladder was manually contoured as an OAR on the planning CT scan following standard anatomical boundaries. Volume was automatically calculated in cm³ by the Eclipse™ Treatment Planning System based on the contour and CT voxel data. All contours underwent standard clinical review as part of plan approval to ensure data consistency.

2) 142 individuals who satisfied the inclusion criteria had their bladder volumes measured. These patients included bladders with volumes of V30, V40, V50, and D2cm^3^, as well as rectums with volumes of V30, V40, V50, and D2cm^3^, and small intestines with volumes of V30, V40, V50, and D2cm^3^. (Bladder V30, V40, V50 indicates the proportion of total bladder volume getting an irradiation dose of 30 Gy, 40 Gy, and 50 Gy, respectively; likewise for rectal and small intestinal volumes). Bladder D2cm^3^ refers to the maximum dose received by a 2cm^3^ volume of the bladder.

3) The bladder volumes of these patients were used to separate them into three groups (<300 mL, 300–500 mL, and >500 mL). These cutoffs were chosen based on the distribution of volumes (range: 54–1193 cm³) to distinguish between low, moderate, and high bladder filling states, and are aligned with common clinical target volumes referenced in pelvic radiotherapy protocols (14, 15). Group A’s bladder volume (V ≤ 300ml); Group B’s bladder volume (300 < V ≤ 500ml); Group C’s bladder volume (V > 500ml). Comparisons were made between the effects of various bladder volumes on the small intestine (V30, V40, V50, D2cm^3^), rectum (V30, V40, V50, D2cm^3^), and bladder (V30, V40, V50, D2cm^3^).

4) GBZ109-2002 “Diagnostic Criteria for Radiation Cystitis” and GBZ111-2002 “Diagnostic Criteria for Radiation Proctitis” were the guidelines followed for grading radiation cystitis and radiation proctitis, and these criteria were applied to all patients. Grading was done over the phone. An analysis was conducted on the frequency of radiation proctitis and cystitis in each group. Classification of radiation-induced cystitis: (1) Mild: characterized by mild symptoms such as urgency, frequency, and pain during urination. Cystoscopy revealed mucosal opacity, congestion, and edema. (2) Moderate: in addition to the aforementioned symptoms, there was capillary dilation hematuria in the bladder mucosa that could recur. Cystoscopy showed mucosal edema, a significant extent of fibrous membranes, capillary dilation, possibly accompanied by ulcers often located on the posterior wall of the bladder triangle or between ureteral folds. (3) Severe: formation of vesicovaginal fistula. Radiation-induced proctitis: Grade I: abdominal pain, anal tingling sensation, loose stools occasionally mixed with blood; Mucosal congestion with hemorrhagic spots and superficial erosion; Grade II: increased urgency after meals or quick bowel movements with frequent defecation; loose stools accompanied by painful defecation often containing blood; Erosion desquamation leading to ulcer formation on the mucous membrane; Grade III: posterior hypersensitivity syndrome causing constipation alternating with loose stools; anal tingling sensation during bowel movements along with bloody stools; Deep ulcer necrosis affecting intestinal wall.

### 2.3 Statistical methods

For all statistical studies, SPSS 26.0 (SPSS Inc., Chicago, U.S.A.) was used. Using the t-test or χ² test, baseline variables such age, bladder volume, FIGO stage, KPS score, and so on were compared between dosing groups. Linear regression was used to quantify relationships between bladder volume and key dose-volume parameters for the bladder (V30, V40, V50, D2cm³), rectum (V30, V40, V50, D2cm³), and bowel bag (V30, V40, V50, D2cm³). Differences in dose-volume parameters were compared using t-tests, and the χ² test was applied to compare the incidence of radiation cystitis and radiation proctitis. For every statistical test, a significance level of *p* < 0.05 was taken into account.

## 3 Results

### 3.1 Patient baseline characteristics

160 patients with cervical cancer in all fulfilled the study’s inclusion requirements. There were 142 patients in the final sample size after 18 patients were eliminated based on the exclusion criteria. Ten patients lost contact during the follow-up period, and ten patients passed away; hence, 122 patients were successfully followed up. The participants ranged in age from 25 to 77 years, with a median age of 53. Three cases were classified as stage IA, twenty-seven as stage IB, twenty-eight as stage IIA, thirty-two as stage IIB, four as stage IIIA, seven as stage IIIB, thirty-four as stage IIIC, six as stage IVA, and one as stage IVB, per the FIGO clinical staging criteria. For each participant, the median Karnofsky Performance Status (KPS) score was 90 (interval: 80-100). IMRT radiotherapy was given to 137 patients, whereas 5 participants underwent TOMO radiation treatment. Based on bladder volume measured at baseline, patients were categorized into three groups: Group A (<300 mL, n=24), Group B (300–500 mL, n=50), and Group C (>500 mL, n=68). A comparison of their baseline characteristics is presented in **Table 1**.

**Table 1:** Clinical profiles of individuals.

Characteristic	Group A (n=24)	Group B (n=50)	Group C (n=68)	*p*
Age (years), Mean	52.96 ± 10.98	52.70 ± 9.998	52.87 ± 10.16	0.9937
FIGO Stage (n)				0.2031
IA	2	0	1	
IB	5	10	12	
IIA	3	13	12	
IIB	6	13	13	
IIIA	0	2	2	
IIIB	3	3	1	
IIIC	5	7	22	
IVA	0	2	4	
IVB	0	0	1	
Bladder volume (cm^3^), Mean	227.9 ± 69.87	408.5 ± 58.79	691.0 ± 159.1	<0.0001
Treatment Technique, n (%)				0.937
IMRT	23 (95.8%)	48 (96%)	66 (97.1%)	
TOMO	1 (4.2%)	2 (4%)	2 (2.9%)	

IMRT, intensity-modulated radiation therapy; TOMO, spiral tomography.

### 3.2 Linear regression analysis of V30, V40, V50 and D2cm^3^ of bladder and rectum with increased bladder volume

**Figure 1:**
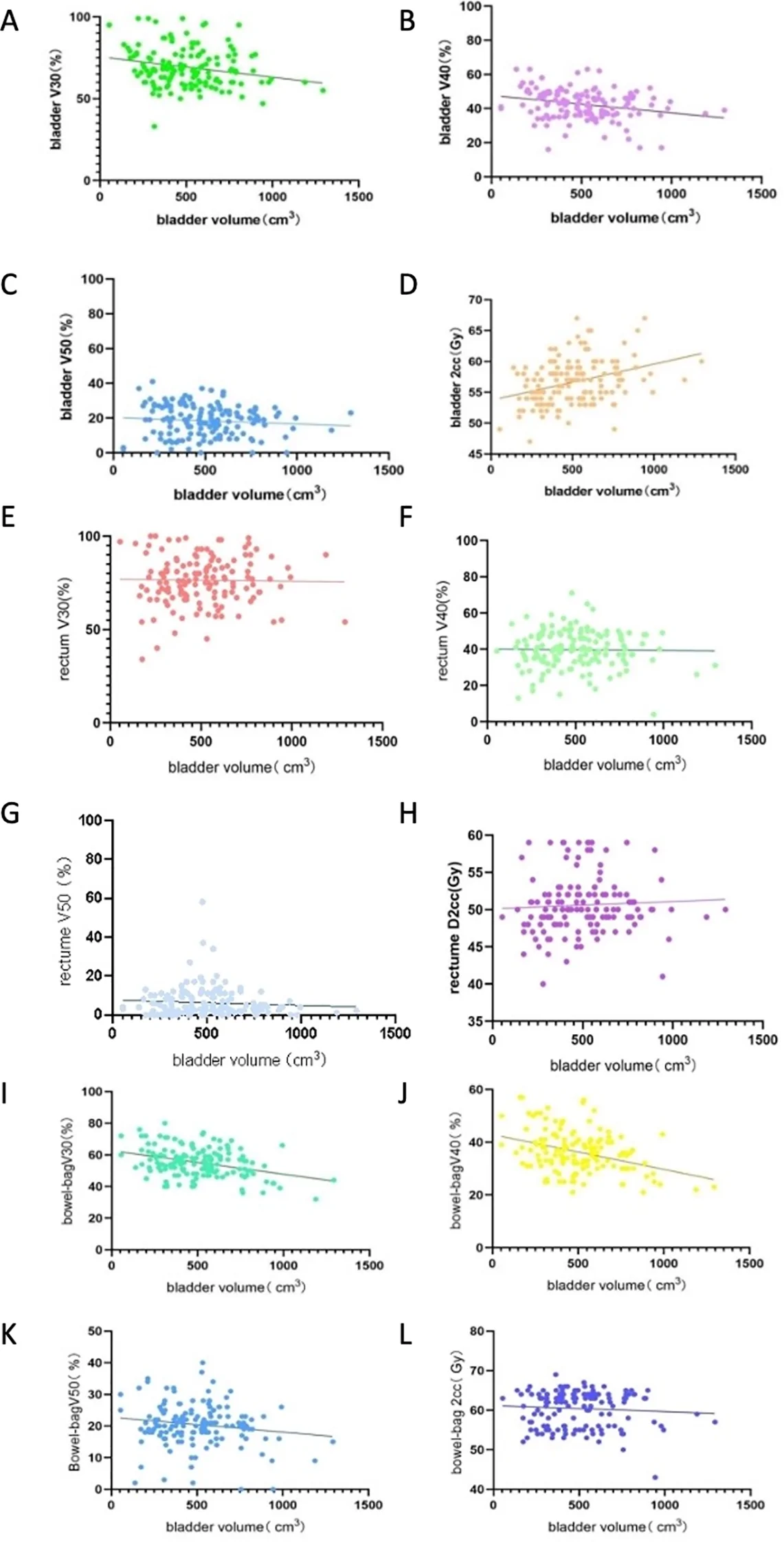
Linear regression analysis of bladder volume versus dose-volume parameters. **(A)** Bladder V30, **(B)** Bladder V40, **(C)** Bladder V50, **(D)** Bladder D2cm³. **(E)** Rectal V30, **(F)** Rectal V40, **(G)** Rectal V50, **(H)** Rectal D2cm³. **(I)** Bowel-bag V30, **(J)** Bowel-bag V40, **(K)** Bowel-bag V50, **(L)** Bowel-bag D2cm³.

The bladder’s capacity varied from 54cm^3^ to 1193cm^3^. Despite the fact that the bladder was filled with water at a set period during the localization process, it was evident that each patient had significant individual variances as well as wide bladder volume gaps. When bladder capacity increased, bladders V30, V40, and V50 all had a downward trend, but bladder D2cm^3^ significantly showed an upward trend (**Figures 1A–D**). Rectal volumes V30, V40, and V50 did not alter considerably with the increase in bladder volume, D2cm^3^ is trending upward (**Figures 1E–H**). The bowel-bag (small intestine)’s V30, V40, and V50 shrank as bladder volume increased, no discernible trend of change in D2cm^3^ (**Figures 1I–L**).

**Figure 2:**
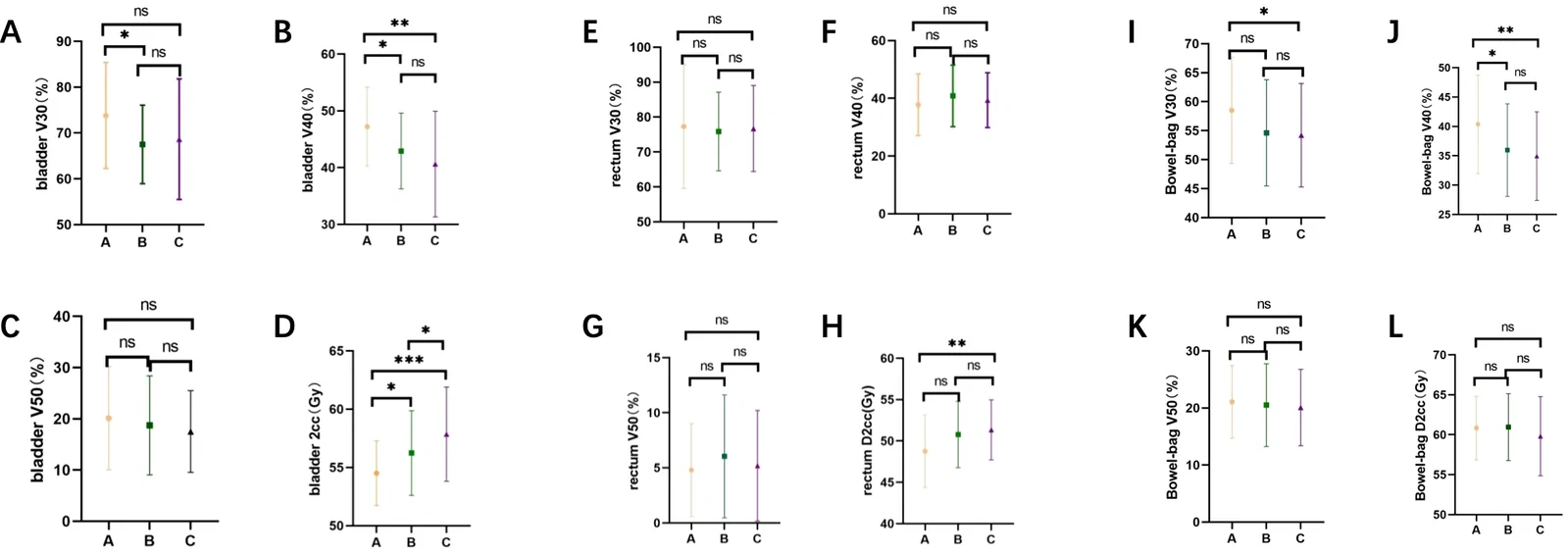
Comparison of dose-volume parameters across bladder volume groups. **(A)** Bladder V30, **(B)** Bladder V40, **(C)** Bladder V50, **(D)** Bladder D2cm³, **(E)** Rectal V30, **(F)** Rectal V40, **(G)** Rectal V50, **(H)** Rectal D2cm³, **(I)** Bowel-bag V30, **(J)** Bowel-bag V40, **(K)** Bowel-bag V50, **(L)** Bowel-bag D2cm³. *P < 0.05, **P < 0.01, ***P < 0.001. ns, not significant.

Comparisons were made between the effects of various bladder volumes (V30, V40, V50, and D2cm^3^) on the bladder (**Table 2** and **Figures 2A–D**). In bladder V30, there was a statistically significant difference between groups A and B (*p* = 0.0102), but not between groups A and C (*p* = 0.0917) or B and C (*p* = 0.5936). In bladder V40, there was no difference between group B and group C (*p* = 0.5936), however there were statistically significant differences between group A and group B (*p* = 0.0121) and between group A and group C (*p* = 0.0022). Bladder V50 did not differ significantly between any of the groups (*p* = 0.4479). Group A and group B (*p* = 0.0418), group A and group C (*p* = 0.0003), and group B and group C (*p* = 0.0273) showed statistically significant differences in bladder D2cm^3^.

**Table 2:** Bladder dose distribution comparison among various cohorts.

Group	Bladder volume	N	Bladder V30 (%)	Bladder V40 (%)	Bladder V50 (%)	Bladder 2cc (Gy)
Group A	V ≤ 300ml	24	73.83 ± 11.56	47.21 ± 6.953	20.13 ± 10.08	54.50 ± 2.782
Group B	300<V ≤ 500ml	50	67.52 ± 8.572	42.92 ± 6.660	18.72 ± 9.668	56.24 ± 3.628
Group C	V> 500ml	68	68.66 ± 13.17	40.63 ± 9.314	17.51 ± 7.964	57.85 ± 4.042
Multiple comparisons		*p* (AB)	0.0102*	0.0102*	0.5656	0.0418*
		*p* (AC)	0.0917	0.0022**	0.2020	0.0003***
		*p* (BC)	0.5936	0.5936	0.4598	0.0273*
Overall comparison		*p*	0.0130*	0.0033**	0.4479	0.0006***

*Statistically significant (p < 0.05). **P < 0.01, ***P < 0.001.

We evaluated the effects of various bladder volumes (V30, V40, V50, and D2cm^3^) on the rectum (**Table 3** and **Figures 2E–H**). Analysis revealed that in all individuals with varying bladder volumes, there was no difference in the rectal V30, V40, and V50 groups (*p* = 0.8897, *p* = 0.4585, *p* = 0.5399). Group A and group C differed in D2cm^3^ (*p* = 0.0053), group B and group C did not differ (*p* = 0.4190), and group A and group B did not differ (*p* = 0.5190).

**Table 3:** Rectal dose distribution comparison among various cohorts.

Group	Bladder volume	N	Rectal V30 (%)	Rectal V40 (%)	Rectal V50 (%)	Rectal 2cc (Gy)
Group A	V ≤ 300ml	24	77.29 ± 17.73	37.79 ± 10.66	4.792 ± 4.222	48.75 ± 4.396
Group B	300<V ≤ 500ml	50	75.84 ± 11.25	40.86 ± 10.67	6.040 ± 5.577	50.78 ± 4.007
Group C	V>500ml	68	76.71 ± 12.32	39.38 ± 9.514	5.191 ± 5.017	51.35 ± 3.627
Multiple comparisons		*p* (AB)	0.6700	0.2507	0.3353	0.0519
		*p* (AC)	0.8595	0.4969	0.7282	0.0053**
		*p* (BC)	0.6964	0.4302	0.3882	0.4190
Overall comparison		*p*	0.8897	0.4585	0.5399	0.0211*

*Statistically significant (p < 0.05). **P < 0.01.

We evaluated the impact of bladder volumes (V30, V40, V50, and D2cm3) on the small intestine (**Supplementary Table 1** and **Figures 2I–L**). All patients’ small intestine V30s revealed a statistically significant difference between groups A and C (*p* = 0.0479), but not between groups A and B (*p* = 0.0925) or B and C (*p* = 0.8128). Group A and B (*p* = 0.0305) and group A and C (*p* = 0.0042) showed statistically significant differences in small intestine V40, but group B and group C showed no difference (*p* = 0.4790). Small intestine V50 did not change between any of the groups (*p* = 0.8228). For all groups, there was no discernible variation in small intestine D2cm3 (*p* = 0.3578).

### 3.3 Analysis of the risk of radiation cystitis and radiation proctitis

Of the 142 patients, 122 were successfully followed up via telephone. Among the remaining 20 patients, 10 were lost to follow-up and 10 had died. Toxicity analysis for radiation cystitis and proctitis was performed on the patients with complete follow-up data, as summarized in **Supplementary Table 2**. The incidence of mild radiation cystitis was highest in Group A (54.17%), compared to Group B (26.20%) and Group C (26.79%). Moderate radiation cystitis was observed only in Group C (5.36%), and no severe cases occurred in any group. The distribution of cystitis grades differed significantly among the three groups (*p* = 0.0396). For radiation proctitis, degree I events were most frequent in Group A (33.33%), followed by Group C (19.64%) and Group B (11.90%). Degree II proctitis occurred at similarly low rates across all groups (Group A: 4.17%; Group B: 7.14%; Group C: 7.14%). No degree III proctitis was reported, and the overall difference in proctitis incidence was not statistically significant (*p* = 0.3385).

## 4 Discussion

Most cervical cancer patients have long-term survival after receiving standard treatment. It is crucial to guarantee their quality of life as a result. Normal tissues like the bladder, rectum, and small intestine are in close proximity to the lesion site during external radiation therapy for cervical cancer. Radiation-induced proctitis and cystitis can be more likely in situations where there are higher radiation doses or more low-dose regions. While satisfying clinical requirements for target coverage, the use of intensity-modulated radiotherapy (IMRT) and tomotherapy (TOMO) has shown to have major benefits in lowering the dose to nearby organs at risk (16–19). Patients are advised to have a full bladder during IMRT and TOMO treatments in order to reduce bladder capacity and safeguard the small intestine (20, 21). However, there is a great deal of diversity in bladder filling among patients due to individual differences and other factors. There is no one ideal bladder filling volume, despite the fact that many research have looked into how varied bladder filling volumes in both local and foreign contexts affect organ-at-risk volumes. Ma and others (22) conducted a study with 166 patients, dividing them into four groups according to the average volume of their bladders throughout radiation treatment (intracavitary brachytherapy and external radiation therapy). Groups A through D are as follows: V < 100 mL, 100 mL ≤ V ≤150 mL, 150 mL < V ≤200 mL, and V > 200 mL. The results of this study showed that bladder volume had a significant impact on target dose delivery for patients with cervical cancer. Patients who had an average bladder volume between 100 and 150 mL had lower incidences of proctitis and cystitis, which is thought to be the ideal range for cervical cancer radiotherapy, which includes intracavitary brachytherapy and external radiation therapy. The average bladder volumes from both internal and exterior exposure were included in this study, however they varied greatly.

According to our research, bladder volumes between 300 and 500 ml have the fewest adverse effects on the bladder, rectum, and small intestine after receiving external radiation therapy for cervical cancer. When comparing bladder volumes of 300–500 ml to <300 ml, the V30 and V40 of the bladder and small bowel were smaller. This was mostly because the increased bladder volume caused the bladder to expand away from the target area and allowed the small bowel to move upward. Whereas bladder volumes >500 ml resulted in greater D2cc in the bladder and rectum, they did not significantly reduce V30 and V40 in the bladder and small intestine when compared to 300–500 ml. This is primarily because the increased bladder volume allowed the anterior rectum wall and posterior bladder wall to be more closely associated with the cervical lesion.

On the contrary, a bladder volume below 300 ml resulted in increased V30 and V40 in both the bladder and small intestine. This also led to a higher occurrence of mild radiation cystitis and mild radiation proctitis. It is worth noting that the incidence of these conditions is closely associated with the low-dose area volume affecting the bladder and small intestine. Conversely, when the bladder volume exceeded 500 ml, there was an increase in D2cc for both the bladder and rectum. This subsequently raised the likelihood of experiencing more than moderate radiation cystitis and radiation proctitis. The occurrence of these conditions can be attributed to receiving a higher maximum dose point on both organs - namely, the bladder and rectum.

There are several limitations to mention. Firstly, this study is retrospective in nature and involved multiple dose-volume comparisons without correction for multiple testing, which increases the risk of false-positive findings. Given the exploratory nature of this analysis, our results should be interpreted as hypothesis-generating and warrant validation in a well-designed prospective cohort with a larger sample size. Secondly, it should be noted that changes in rectal volume may potentially impact the experimental data. Our previously published work showed that for external irradiation in patients with cervical cancer, a rectal volume of 40–70 ml is associated with the most favorable outcomes (23). However, rectal volume was not systematically controlled or recorded in the present study, and its potential impact on bladder-based dosimetry cannot be ruled out. Future studies should include both bladder and rectal volume monitoring to more comprehensively assess organ-at-risk sparing. Thirdly, our analysis is based on bladder volume from a single planning CT. As bladder filling varies during treatment, our dosimetric data reflect the planned scenario rather than the delivered dose to a dynamically changing organ. Fourthly, aligning bladder volume accurately with the patient’s position during treatment poses challenges, though daily image guidance could mitigate this. Fifthly, the inability to perform stratified analyses by treatment intent (definitive vs. postoperative) or disease stage, both of which may influence target volume and dosimetric outcomes, underscores the need for future studies to explore these variables in larger, more balanced cohorts. Finally, the overwhelming predominance of IMRT (96.5% of patients) over TomoTherapy (3.5%) precludes a meaningful comparative analysis of treatment techniques as a potential confounding variable. Despite these limitations, our findings provide valuable insight into the relationship between bladder volume and organ-at-risk dosimetry in routine clinical practice.

## 5 Conclusion

For patients with cervical cancer undergoing external irradiation, a bladder volume of 300ml to 500ml is ideal.

## Data Availability

The raw data supporting the conclusions of this article will be made available by the authors, without undue reservation.
