# Correction: Effect of bladder volume on dose of exposure to dangerous organs and incidence of cystitis and enteritis in patients with cervical cancer after external radiotherapy

**DOI:** 10.3389/fonc.2026.1959912

**Published:** 2026-08-18

**Authors:** Yanjiao Wu, Xiaoying Xue, Linlin Su, Yibin Liu

**Affiliations:** 1 Department of Radiotherapy, The Second Hospital of Hebei Medical University, Shijiazhuang, Hebei, China; 2 Department of Gynecology, The Second Hospital of Hebei Medical University, Shijiazhuang, Hebei, China

**Keywords:** bladder volume, cervical cancer, external pelvic irradiation, organ at risk, real-world data

The funder “Medical Science Research Project of Hebei, 20241168” to “Yanjiao Wu” was erroneously omitted.

The original version of this article has been updated.

