# Supplementary material for: Effect of bladder volume on dose of exposure to dangerous organs and incidence of cystitis and enteritis in patients with cervical cancer after external radiotherapy
Source: Front Oncol. 2026 Mar 20;16:1760076. doi: 10.3389/fonc.2026.1760076 (PMC13047064; doi:10.3389/fonc.2026.1760076)
Supplement: Supplementary file 1 [file Table1.docx]

**Supplementary Materials**

**Supplementary Table 1** Small bowel dose distribution comparison among various cohorts

| Group | Bladder volume | N | Small bowel V30 (%) | Small bowel V40 (%) | Small bowel V50 (%) | Small bowel 2cc (Gy) |
| --- | --- | --- | --- | --- | --- | --- |
| Group A | V≤300ml | 24 | 58.50±9.151 | 40.38±8.397 | 21.08±6.372 | 60.83±3.975 |
| Group B | 300＜V≤500ml | 50 | 54.62±9.169 | 35.96±7.892 | 20.50±7.257 | 60.96±4.189 |
| Group C | V＞ 500ml | 68 | 54.22±8.929 | 34.94±7.558 | 20.09±6.693 | 59.82±4.959 |
| Multiple comparisons | | *p* (AB) | 0.0925 | 0.0305* | 0.7377 | 0.9019 |
|  |  | *p* (AC) | 0.0479* | 0.0042** | 0.5278 | 0.3707 |
|  |  | *p* (BC) | 0.8128 | 0.4790 | 0.7506 | 0.1921 |
| Overall comparison | | *p* | 0.1283 | 0.0151* | 0.8228 | 0.3578 |

*Statistically significant (p < 0.05)

**Supplementary Table 2**. Comparison of the occurrence rates of cystitis and proctitis among various groups

| Group | N (all) | N (actual) | Mild Radiation cystitis | Moderate Radiation cystitis | Severe Radiation cystitis | *p* |
| --- | --- | --- | --- | --- | --- | --- |
| Group A | 24 | 24 | 13 (54.17%) | 0 (0.00%) | 0 (0.00%) | 0.0396* |
| Group B | 50 | 42 | 11 (26.20%) | 0 (0.00%) | 0 (0.00%) |  |
| Group C | 68 | 56 | 15 (26.79%) | 3 (5.36%) | 0 (0.00%) |  |
| Group | N (all) | N (actual) | Radiation proctitis Degree I | Radiation proctitis Degree II | Radiation proctitis Degree III | *p* |
| Group A | 24 | 24 | 8 (33.33%) | 1 (4.17%) | 0 (0.00%) | 0.3385 |
| Group B | 50 | 42 | 5 (11.9%) | 3 (7.14%) | 0 (0.00%) |  |
| Group C | 68 | 56 | 11 (19.64%) | 4 (7.14%) | 0 (0.00%) |  |

*Statistically significant (p < 0.05)
